# Multimodal Evaluation of Mental Workload and Engagement in Upper-Limb Robot-Assisted Motor Tasks

**DOI:** 10.3390/s26030922

**Published:** 2026-01-31

**Authors:** Camilla Zanco, Marta Mondellini, Matteo Lavit Nicora, Matteo Malosio, Giovanni Tauro, Giovanna Rizzo, Alfonso Mastropietro

**Affiliations:** 1 Institute of Intelligent Industrial Technologies and Systems for Advanced Manufacturing, National Research Council, 20133 Milan, Italy; camillazanco@cnr.it (C.Z.); giovanna.rizzo@cnr.it (G.R.); 2 Department of Psychology, Catholic University of Milan, 20123 Milan, Italy; 3 Institute of Intelligent Industrial Technologies and Systems for Advanced Manufacturing, National Research Council, 23900 Lecco, Italy; marta.mondellini@cnr.it (M.M.); matteo.lavitnicora@cnr.it (M.L.N.); matteo.malosio@cnr.it (M.M.); giovanni.tauro@stiima.cnr.it (G.T.); 4 Rehabilia Technologies SRL, 20123 Milano, Italy; 5 Industrial Engineering Department, University of Bologna, 40126 Bologna, Italy

**Keywords:** electroencephalography(EEG), electrocardiogram (ECG), robot-assisted rehabilitation, engagement, mental workload

## Abstract

Patient engagement and mental workload (MWL) are often overlooked when optimising robotic-assisted rehabilitation, despite their potential impact on its effectiveness. This study aims to propose a multimodal approach to assess MWL and engagement, using electrophysiological signals and questionnaires, to explore their modulation across different assistance modalities and engaging strategies. Thirty healthy subjects were enrolled and performed repetitive upper-limb movements with a robotic device under three assistance modalities (active, passive, semi-assisted) while listening to a 1 Hz auditory stimulus (metronome or music). Electroencephalography, Electrocardiogram, the NASA Task Load Index, and the Short Stress State Questionnaire were used to assess objective and perceived MWL and engagement. Engagement increased significantly in the music condition, whereas MWL showed no significant change. The passive modality was perceived as significantly less demanding and less engaging compared to active and semi-assisted conditions. Although EEG objective indicators did not vary across modalities, the ECG objective metric was modulated significantly in agreement with the subjective measures. Overall, the auditory stimulus significantly influenced engagement, and assistance levels affected both perceived mental demand and engagement. The proposed multimodal approach is sensitive to both engagement and MWL constructs, highlighting the potential for adaptive rehabilitation systems designed to maintain engagement, prevent overload or monotony, and ultimately support better functional outcomes over the long term of robotic training.

## 1. Introduction

Upper-limb motor rehabilitation is crucial for regaining functional independence after neurological diseases, as arm and hand impairments significantly limit the performance of everyday activities, including reaching, grasping, and object manipulation. In this context, robotic devices have emerged as pivotal tools in neurorehabilitation, enabling precise, intensive, repetitive, and adaptable therapy tailored to each patient’s needs. Evidence from several studies demonstrates that robot-assisted, task-specific, and intensive training can significantly enhance motor recovery in different neurological disorders [1,2,3]. Moreover, tailoring the level of robotic assistance to the patient’s residual physical capacity can help improve rehabilitation outcomes [4,5]. Such adaptive assistance strategies can also be integrated into bilateral training approaches, which have demonstrated specific neurophysiological benefits for motor recovery [6,7].

Typically, studies evaluating the effects of motor rehabilitation focus primarily on motor performance, often overlooking the broader cognitive, psychological, and mental states of the patient. Indeed, the most common studied measures of motor upper limb recovery (e.g., the Fugl–Meyer Assessment, the Functional Independence Measure, the Action Research Arm Test, the Box and Block Test, the modified Ashworth Scale and the Wolf Motor Function Test) assess different aspects of motor recovery, such as movement quality, dexterity, strength and task-related ability [8,9]. In this context, electrophysiological measures such as the electroencephalography (EEG) have also been used to evaluate [10] and predict rehabilitation outcomes, although still mainly in relation to motor recovery [11,12].

However, beyond a patient’s residual physical capacity to perform motor tasks, other factors can affect performance and clinical outcomes. Indeed, mental workload (MWL) and engagement can play a key role in facilitating neuroplastic mechanisms and promoting functional improvement [13,14]. MWL can be defined as “the degree of activation of a finite pool of resources, limited in capacity, while cognitively processing a primary task over time, mediated by external stochastic environmental and situational factors, as well as affected by definite internal characteristics of a human operator, for coping with static task demands, by devoted effort and attention [15]. In this specific context, MWL can be viewed as the user demand required by the assistive device and can have an impact on the patient’s experience [16]. For example, when the task demand during therapy is set too high, patients may experience stress or frustration, which reduces training effectiveness. Conversely, when the challenge is too low or the activity becomes overly repetitive, boredom and disengagement can occur [17,18,19]. Both cases lead to poorer performance and reduced effectiveness of the rehabilitation process. For this reason, monitoring MWL is crucial to appropriately balance task demands, ensuring that rehabilitation activities remain neither overly demanding nor insufficiently stimulating, to enhance rehabilitation success.

The optimal balance between task difficulty and the patient’s skill level leads to the ’Flow State’, characterized by maximum engagement and enjoyment [20]. This high level of engagement is a critical factor, as it increases patients’ motivation to participate in the therapeutic activity for its own sake, thereby enhancing their adherence to therapy. This leads patients to train more frequently and consistently, which in turn further enhances the likelihood of functional recovery [21]. In this direction, numerous studies have explored strategies to enhance patient engagement. These include the use of exergames, which combine visual stimuli to provide patients with interactive exercises [22,23], as well as auditory stimuli (e.g., rhythmic auditory stimuli and music). For instance, the use of rhythmic auditory stimuli to encourage movement and long-term functional improvements in patients with motor impairments has shown promising results in motor rehabilitation outcomes [24,25]. In the past years, an increasing number of clinical studies provided valuable insights into the benefits of music therapy, showing that musical rhythms can serve as a continuous time reference to patients with movement-related disorders [24], and can act as an engaging factor during rehabilitation sessions [26,27].

Since workload, motivation, and emotional factors play a crucial role in determining rehabilitation outcomes, their systematic assessment during rehabilitation sessions may be essential to personalize and optimize the effectiveness of the entire rehabilitative process. Moreover, a joint evaluation of engagement and mental workload is important for better characterizing how patients interact with assistive robots across different tasks and motivational conditions.

In this context, monitoring of physiological signals has emerged as a promising approach for objectively assessing the patient’s psychological and cognitive state during rehabilitation. Electrophysiological biomarkers can reflect underlying emotional and cognitive processes, eliminating the need for active patient cooperation [28]. Frequently used physiological measurements include electrocardiography (ECG), electrodermal activity (EDA), EEG, respiration and skin temperature, which together offer insights into autonomic regulation, engagement, and MWL. Consistent findings indicate that increased task demand or mental effort is associated with elevated heart rate and decreased average value of Normal-to-Normal Intervals (AVNN) [29,30], lower values of heart rate variability (HRV) parameters (e.g., Root Mean Square of Successive Differences (RMSSD) and Standard Deviation of Normal-to-Normal intervals (SDNN)) [31,32], higher skin conductance [32,33,34], higher values of the EEG-MWL index (MWLI) [35,36,37], faster and less variable respiration [31,38], and lower skin temperature [39,40].

Regarding the objective assessment of engagement, several studies have computed an EEG-based engagement index (EI) across various contexts and applications. For example, in video game play, there is evidence that EI can discriminate users’ engagement across different tasks or scenarios [41]. Additionally, autonomic-related signals, such as EDA and HRV, exhibited synchronized responses to specific stimuli, corresponding to higher levels of attentional engagement [42].

In the field of robot-assisted motor rehabilitation, recent studies have integrated some of these psychophysiological measures to evaluate user’s state during rehabilitation sessions. However, while engagement has received increasing attention [43,44], the assessment of MWL specifically during robotic rehabilitation remains largely unexplored.

Nevertheless, subjective variables such as perceived mental effort and engagement represent crucial aspects of the user experience that cannot be fully captured through physiological indicators alone. These dimensions play a key role in shaping how patients interact with rehabilitation tasks and, ultimately, in influencing recovery outcomes. To assess these subjective components, self-report instruments such as the NASA Task Load Index (NASA-TLX) [45] and the Short Stress State Questionnaire (SSSQ) [46] are commonly employed. These tools provide insight into how people consciously perceive the difficulty, effort, and emotional quality of an experience, including rehabilitation tasks [47]. However, self-reports are inherently influenced by subjective interpretation, awareness, and communication ability, which can be compromised in clinical populations.

For this reason, by integrating electrophysiological measurements with self-report questionnaires, recent studies have provided a more holistic representation of the patient’s experience during rehabilitation [28,43,48,49]. This complementary approach helps identify how physiological responses correspond to perceived workload and emotional states, paving the way for user-centered adaptive rehabilitation protocols that can dynamically tailor assistance based on the patient’s psychophysiological condition and feedback [50].

Within this context and to the best of our knowledge, no previous studies have examined how MWL and engagement vary across different levels of robotic assistance and sensory stimulation modalities (e.g., music during exercise) while integrating objective electrophysiological measures with validated self-reports in robot-assisted upper-limb motor rehabilitation. In fact, most existing research either focuses exclusively on assistance levels [51,52,53] or on user engagement [43,54], and others rely solely on isolated physiological signals [50,55], without jointly investigating how all these dimensions interact during motor training.

This work addresses this gap by proposing a multimodal framework to evaluate MWL and engagement modulation across different levels of robot-assisted movement and auditory-stimulation modalities, complementing objective measures (based on EEG and ECG signals) with subjective assessments.

## 2. Materials and Methods

### 2.1. Participants

In total, 30 healthy individuals were recruited (21 males, 9 females; see Table 1 for more details). Before the experimental session began, all volunteers were briefed on the protocol and signed a written informed consent form. The study was approved by the Ethical Committee of the National Research Council of Italy (Approval No. 0083017, 19 March 2025) and was conducted in accordance with the Declaration of Helsinki, ensuring adherence to ethical standards for research involving human subjects.

### 2.2. Set-Up

The instrumentation used for the study included an upper-limb robotic device and an EEG-ECG acquisition system. These are described in more detail in the following paragraphs.

#### 2.2.1. Robotic Device

**Figure 1:**
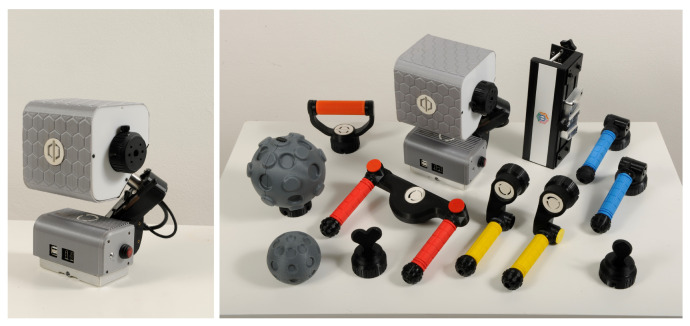
PhiCube’s central unit and handles.

The robotic device used in this study is “PhiCube”, a patented compact and modular robotic device designed to combine motor training with cognitive, visual, and attentional activities [56,57]. It consists of a robotic central unit, a table-mounting mechanism, and a set of handles with different shapes and functions (Figure 1). The central unit is equipped with two motorized and sensorized interfaces and an orientation mechanism that allows the alignment of the movement axes along the main body planes (sagittal, frontal, vertical). Thanks to this modular solution, the device can support a wide range of upper-limb movements, including bilateral tasks. Three assistance modalities that differ in the level of participant motor involvement are allowed: (i) active; (ii) passive, in which the user does not voluntarily execute any movement; and (iii) semi-assisted mode, in which the movement of one limb is generated based on the contralateral one. The device is connected to a monitor displaying a dedicated graphical user interface (GUI). The GUI can be used to set the desired level of motor assistance/resistance or to activate the semi-assisted mode. Additionally, the application offers a series of training activities designed to contextualize the selected exercise (e.g., exergames).

**Figure 2:**
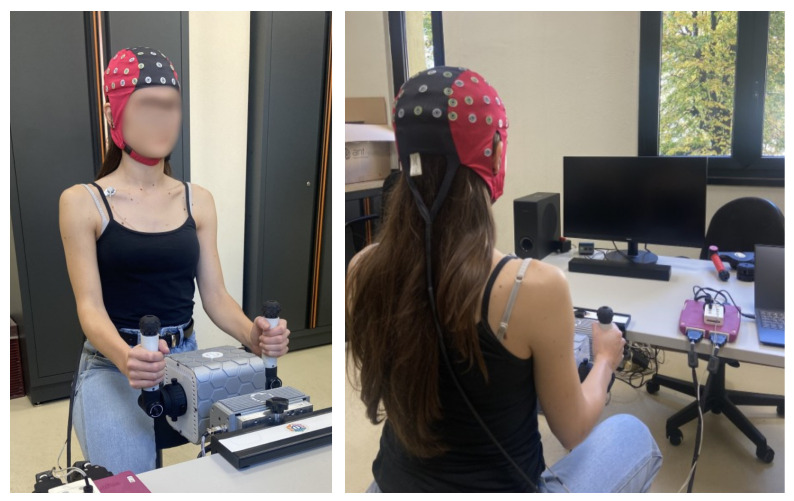
Experimental set-up including the upper-limb rehabilitation robot and the physiological data acquisition device.

For this specific study, PhiCube’s central unit was aligned with the participant’s sagittal plane and two independent lever accessories were installed onto the plug-and-play interfaces to allow counterphase bilateral movements of wrist, elbow, and shoulder (Figure 2).

#### 2.2.2. Electrophysiological Signal Acquisitions

EEG was collected using a 64-channel eego™ mylab system (ANT Neuro, Hengelo, The Netherlands), equipped with a 24-bit amplifier, a standard EEG cap (Waveguard™ original, ANT Neuro, Hengelo, The Netherlands) with 64 active Ag/AgCl electrodes (Figure 2) positioned according to the international 10–10 system and the eego software (Version: 1.10.2.45890) to manage the acquisition. First, the nasion–inion distance was measured to identify the central point of the scalp. Then, the EEG cap was positioned with the Cz electrode placed at the midpoint of the nasion–inion distance. Finally, the conductive gel was applied to reduce the electrode-skin impedance below 30 kΩ throughout the recording session. The EEG signal was recorded at a sampling rate of 500 Hz across 62 channels (mastoid electrodes were excluded) and the online reference was set at the centroparietal midline electrode (CPz). The electrocardiographic (ECG) signal was acquired from the bipolar sensor input of the EEG amplifier. Two surface electrodes (30 × 24 mm, Cardinal Health™ Kendall™, Waukegan, IL, USA) were placed on the participant’s torso to capture a single bipolar lead. Specifically, the negative electrode was positioned at the center of the right clavicle, whereas the positive electrode was located on the lowest left rib, along the midaxillary line. Both signals were recorded as differential voltage, with EEG amplitudes in the µV range and ECG amplitudes in the mV range. Despite originating from ionic currents in tissue, the amplifiers’ high input impedance results in negligible current flow, and no current waveform was considered. The time needed to set the EEG and ECG sensors ranged from 15 to 45 min, depending on how easily the electrode–skin impedance could be brought below the desired value.

### 2.3. Protocol

The experimental protocol consisted of five phases. Firstly, a 5 min resting phase (Rest) was performed to acquire baseline data, followed by four different movement phases (Task). During the Rest, the subject had to stare at a white cross on a black background in the monitor in front of them, holding the handles of PhiCube.

The Task consisted of moving the two handles in counterphase (one arm ahead, the other backwards) repetitively following an auditory stimulus at 1 Hz. The experiment involved three robotic assistance modes cited previously (active, passive, and semi-assisted) and two types of auditory stimuli (metronome and music), which were combined in four distinct task conditions: Active with Metronome (AME), Active with Music (AMU), Passive with Metronome (PME), and Semi-assisted with Metronome (SME). In particular, the three assistance modalities differed in the level of participant motor contribution: (i) in AME/AMU the volunteer was required to autonomously perform muscular contraction in order to move both handles; (ii) in PME the participant was instructed to grasp the handles, relax both upper limbs and let PhiCube mobilize them without anticipating or resisting the movement; (iii) in SME the participant was asked to perform the movement with the dominant limb while keeping the other one passive. In SME, PhiCube performed the movement on the non-dominant side autonomously as a counterphase mirror of the dominant one. The auditory stimuli consisted of (i) a metronome sound at 1 Hz frequency (in AME, PME and SME tasks); (ii) a music track matched to the same frequency as the metronome (in AMU task).

The four modalities (i.e., AME, AMU, SME and PME) were presented in a random order across subjects, but with AME and AMU always executed randomized in close proximity to each other. This choice is motivated by the fact that switching between very different modalities (e.g., active–passive–active) can require cognitive adjustments that affect performance and might induce confusion and potential execution errors. Therefore, keeping the two versions of the active assistance together would have reduced such transition effects.

**Figure 3:**
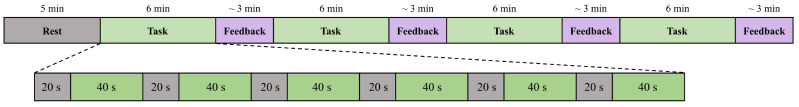
Schematic of the protocol followed by each participant.

Each Task involved six rest runs (each lasting 20 s) alternating with six movement runs (each lasting 40 s) (Figure 3). A countdown of three seconds appeared on the screen before the next run began, notifying the subject of the change. During the experimental activities, the PhiCube application (i.e., the screen in front of the participant) only showed a black background during movement runs and a black background with a white cross in the center of the screen during rest runs. At the end of each Task, the participant was asked to complete the questionnaires on perceived MWL and engagement (more details of the questionnaires can be found in Section 2.5). This feedback session lasted approximately 3 min (Figure 3).

All experimental sessions were conducted in the same controlled laboratory environment, designed to minimize noise sources that could disrupt the participants’ performance. Moreover, the subject was asked to keep the head as still as possible and not to talk during Rest or Tasks to limit noise in the EEG signal.

### 2.4. Objective Assessment of Mental Workload and Engagement

**Figure 4:**
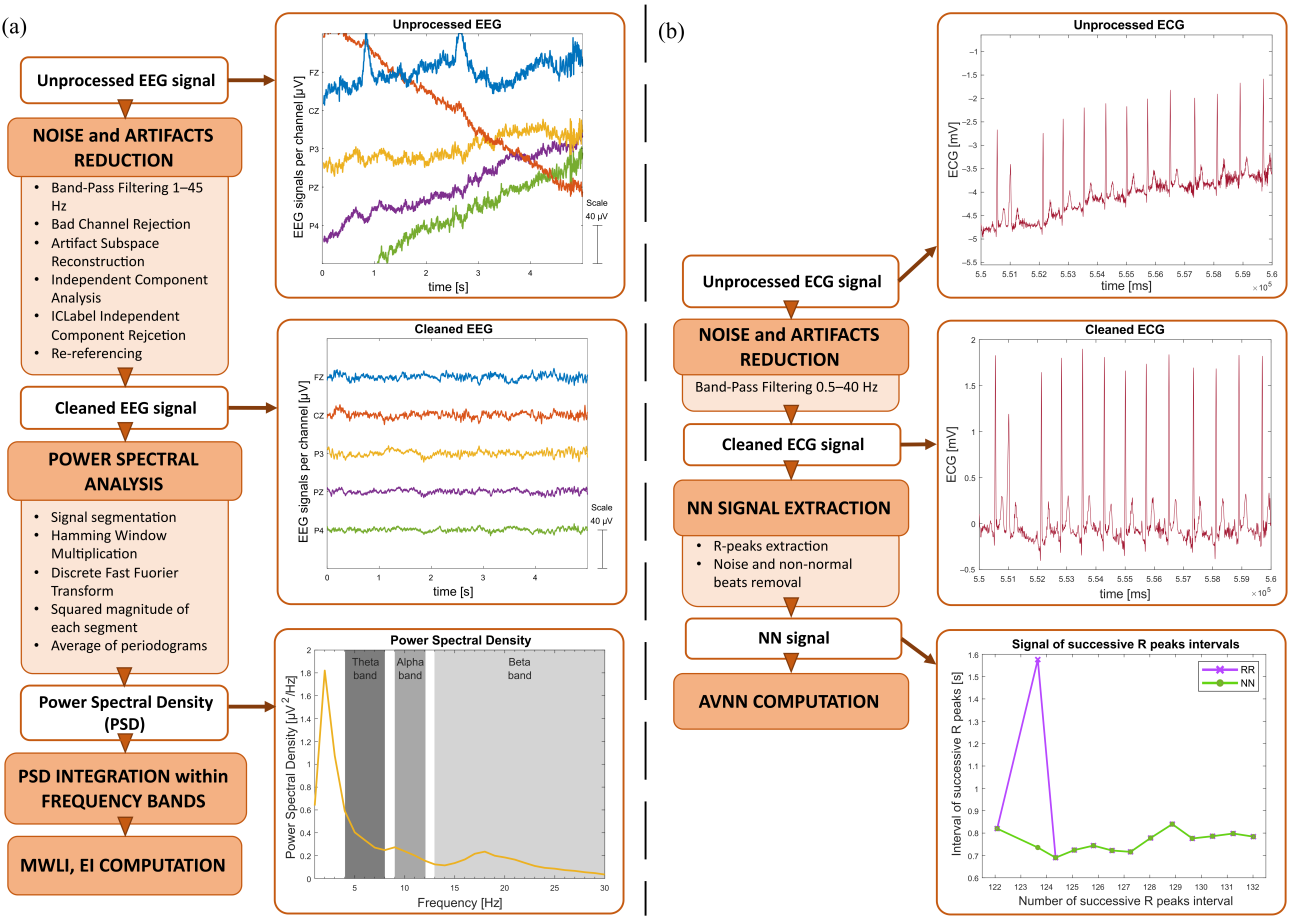
Schematic diagram of the pre-processing and analysis steps followed to compute the objective parameters. In panel (**a**) the pipeline adopted to process the EEG signal and example plots of the signals of the five used electrodes (Fz, Cz, P3, Pz, P4) at the key stages of the workflow: in the upper figure a plot of the unprocessed EEG; in the middle, the cleaned EEG; and at the bottom, the power spectral density of the electrode P3 highlighting the three investigated frequency bands (theta, alpha, beta). In panel (**b**) the steps taken to analyze the ECG signal supported by graphic representation of the ECG signal at the key stages of the workflow: in the upper figure, the unprocessed ECG; in the middle, the cleaned ECG; and at the bottom, the RR and the Normal-to-Normal interval (NN) signals.

The EEG analysis was performed using MATLAB (R2024a, The MathWorks) and EEGLAB toolbox [58], whereas the ECG signal was mainly analyzed using the Physionet toolbox [59,60]. Figure 4a,b schematically explains the steps performed for EEG.

#### 2.4.1. EEG

**Figure 5:**
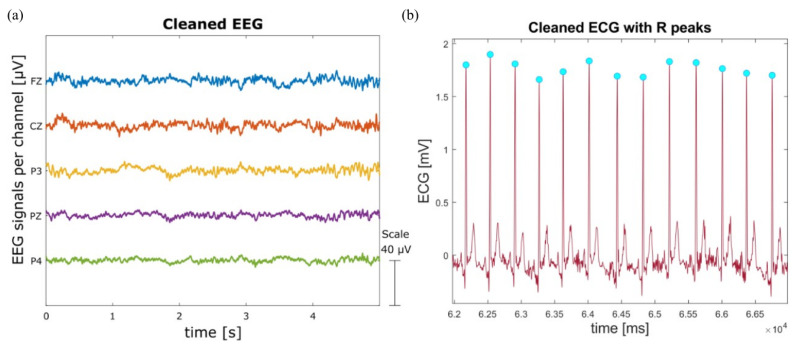
Graphic representation of the physiological filtered signals used to compute the objective parameters evaluated. In panel (**a**), the EEG signal of the electrodes used in the computation of the objective parameters (Fz, Cz, P3, Pz, P4); in panel (**b**), the ECG signal, with R peaks highlighted in light blue dots.

The EEG signal was pre-processed as previously reported in [61]. First, the data were filtered using a Hamming-windowed sinc Finite Impulse Response band-pass filter (1–45 Hz). Secondly, artifact channels were rejected using the joint probability of the average log-power across recorded electrodes, as proposed in [62]: channels with probabilities exceeding three standard deviations from the mean were removed. After this, the Artifact Subspace Reconstruction algorithm [63] was applied to interpolate segments containing artifacts, defined as bursts with variances exceeding fifteen standard deviations. Following this, Independent Component Analysis was performed using the extended Infomax algorithm [64] to identify and remove artifacts, such as eye movements and electrocardiographic signals, which typically overlap with neural activity in EEG data. Then, ICLabel [65] was used to automatically classify components, and those with a probability below 40% of being brain sources were discarded. Finally, the bad channels were removed and interpolated using a spherical function based on neighbouring artifact-free channels, and the EEG signals were re-referenced to the average of all channels (Figure 5a).

Afterwards, the MWLI and the Engagement Index (EI) were computed to address MWL and engagement, respectively. Firstly, the Task sessions were discarded, with the six 20 s rest periods, to keep only the movement part. On each movement run, the Welch method was applied to estimate the power spectral density between 0 and 45 Hz [66]. The EEG signal was divided into 50% overlapping segments of 1 s length, and each segment was multiplied by a Hamming window. Then, the discrete Fourier transform was applied to each windowed segment, and the squared magnitude of the result was computed to obtain the periodogram. Periodograms were subsequently averaged across all segments within each movement run to derive the power spectral density corresponding to each experimental block. Next, the absolute band power for each channel was calculated by integrating the power spectrum within theta (4–8 Hz), alpha (9–12 Hz) and beta (13–30 Hz) frequency bands. Finally, the MWLI was computed, for each experimental block, as the ratio between the theta absolute power at the frontal midline electrode (*θ_Fz_*) and the alpha absolute power at the parietal midline electrode (*α_Pz_*) [61,67]. This measure, which reflects changes in the EEG signal power spectral density in the theta and alpha frequency bands, is therefore used to assess the cognitive effort. To compute the EI, the sum of the absolute power of each frequency band at the central midline, parietal midline, left and right electrodes (Cz, Pz, P3 and P4) was calculated. Subsequently, the EI was computed as the ratio between the beta power ($\beta_{Cz,Pz,P3,P4}$) and the sum of alpha power ($\alpha_{Cz,Pz,P3,P4}$) and theta power ($\theta_{Cz,Pz,P3,P4}$) [68,69]. This formulation of the EI enables the assessment of an individual’s engagement while performing a specific activity.

#### 2.4.2. ECG

The ECG signal was also pre-processed prior to performing time-domain HR analysis. Firstly, a bandpass (0.5–40 Hz) Hamming-windowed sinc FIR filter was applied. From the clean signal, only Rest and movement blocks for each Task were kept. On these signals, R peaks were extracted using Physionet functions (Figure 5b). Then, RR interval data were processed in preparation for further analysis: noise and non-normal beats were removed and replaced with the cubic spline interpolation method, obtaining the Normal-to-Normal interval signal (NN). This processed time-series was then used to compute the AVNN, representing the mean duration of normal heartbeats, which is negatively related to MWL [29,30]. Each 40 s task window was selected to compute the AVNN, and the resulting values were averaged on each experimental session to have a single value for the Rest and for each Task. It was not possible to compute any further ECG-based parameters or perform an HRV analysis, as the movement runs in the protocol were insufficiently long.

### 2.5. Subjective Assessment of Mental Workload and Engagement

At the end of each Task, participants were asked to complete the NASA-TLX [45] and the Engagement subscale of the SSSQ [46] questionnaires.

#### 2.5.1. NASA-Task Load Index Scale

The NASA-TLX scale is a widely used subjective assessment tool designed to evaluate the perceived cognitive workload during or after completing a specific task [45]. It consists of six dimensions: Mental Demand, Physical Demand, Temporal Demand, Performance, Effort, and Frustration. Participants are asked to rate each dimension using a 21-point continuous scale ranging from 0 to 20. An overall subjective score (hereafter, NASA-TLX Overall) is obtained by summing the single scores of the six dimensions.

#### 2.5.2. Short Stress State Questionnaire

The Short Stress State Questionnaire [46] is a self-assessment instrument developed to rapidly measure subjective stress levels associated with performing cognitively or emotionally demanding tasks. The questionnaire consists of 24 items that assess three key dimensions of momentary psychological state: Engagement, Distress, and Worry. In this study, we selected the Engagement scale, which reflects the individual’s degree of positive involvement, energy, and motivation during the activity. Engagement subscale measures dimensions such as concentration, readiness for action, sense of control, and confidence in self-abilities. High scores on the Engagement scale indicate an optimal state of activation and participation, associated with adequate levels of mental energy and involvement; conversely, lower scores suggest fatigue, disengagement, or a decline in concentration. The instrument uses a 5-point Likert scale, and scores for each subscale are obtained by averaging the responses to the corresponding items.

### 2.6. Data Analysis

All statistical analyses were done using R (R 4.4.1, R Foundation for Statistical Computing, Vienna, Austria) within the RStudio environment (Version: 2024.12.0+467).

To allow comparison between objective and subjective indexes and to account for inter-subject variability, all objective indexes of each Task were subject-wise normalized by dividing the Task value for its corresponding Rest value (Task/Rest).

To verify normality, the Shapiro–Wilk test was applied to each index for each Task independently. If the *p*-value was smaller than 0.05, the null hypothesis was rejected, meaning that the data were not normally distributed. On the contrary, if the *p*-value was greater than 0.05, then the null hypothesis could not be rejected and graphical solutions were considered to determine whether the data were Gaussian or not. In particular, both the histogram and the Quantile–Quantile Plot were analyzed.

To separately compare the different levels of robot assistance and the effect of the different acoustic stimuli on the subject, data were grouped as follows: AME, PME, and SME (Assistance Levels), and AME and AMU (Auditory Stimuli). As all tested indices showed non-Gaussian distributions for each Task and the data were dependent due to repeated measures, the following analyses were performed. For the Assistance Levels, the Friedman rank-sum test was first performed for each objective and subjective index. If any statistically significant difference was found (*p* < 0.05), post hoc Nemenyi’s All-Pairs Comparisons Test was performed. Instead, for the Auditory Stimuli, only the Wilcoxon pairwise test was applied as there were just two conditions to compare.

Additionally, to quantify the relationship between physiological and subjective variables, we performed repeated measures correlation analysis [70] for the Assistance Levels and for the Auditory Stimuli, separately.

## 3. Results

### 3.1. Objective Measures

**Figure 6:**
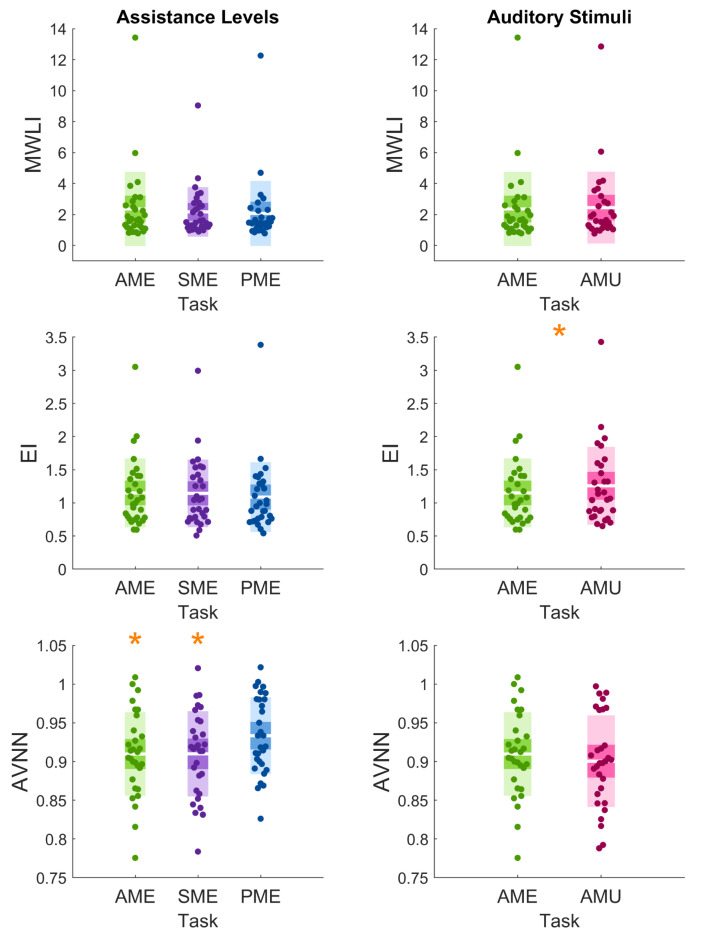
Scatter distributions of the objective physiological indexes Mental Workload Index (MWLI) (**top row**), Engagement Index (EI) (**middle row**), and Average Value of Normal-to-Normal intervals (AVNN) (**bottom row**) across Tasks for the Assistance Levels (**left column**) and the Auditory Stimuli (**right column**). Each dot represents an individual participant’s value. The white horizontal line indicates the mean, the darker shaded band represents the 95% confidence interval of the mean, and the lighter colored area corresponds to one standard deviation. Asterisks denote statistically significant differences between conditions, based on Nemenyi post hoc for the Assistance Levels and Wilcoxon pairwise comparison for the Auditory Stimuli.

Regarding the Assistance Levels, the Friedman test showed a significant difference for the AVNN index. Post hoc pairwise comparison highlighted a significant increase from both AME to PME (*p* < 0.001) and from SME to PME (*p* < 0.01) (see Figure 6 and Table 2). The MWLI and the EI did not show any significant difference between modalities. Regarding the Auditory Stimuli, the EI highlighted a significant increase in the Task with music compared to the metronome sound (*p* < 0.001, see Figure 6 and Table 2). Neither the MWLI nor the AVNN revealed any significant difference across the two auditory modalities.

### 3.2. Subjective Measures

**Figure 7:**
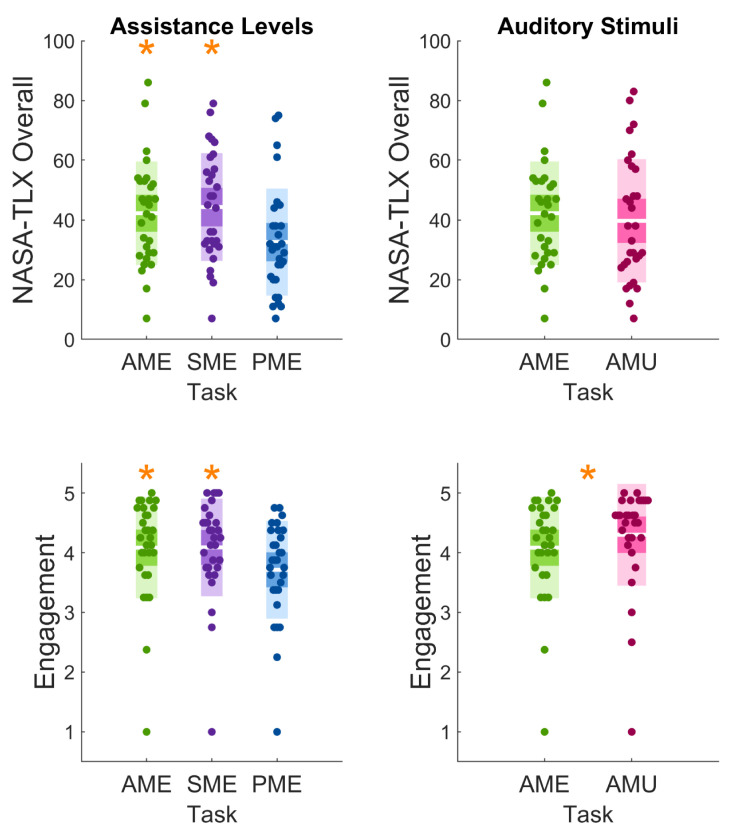
Scatter distributions of the subjective self-assessed indexes NASA-TLX Overall (**top row**) and Engagement (**bottom row**) across Tasks for the Assistance Group (**left column**) and the Auditory Stimuli (**right column**). Each dot represents an individual participant’s value. The white horizontal line indicates the mean, the darker shaded band represents the 95% confidence interval of the mean, and the lighter colored area corresponds to one standard deviation. Asterisks denote statistically significant differences between conditions, based on Nemenyi post hoc for the Assistance Levels and Wilcoxon pairwise comparison for the Auditory Stimuli.

In the Assistance Levels, both the NASA Task Load Index (NASA-TLX) Overall and the Engagement indices revealed a significant difference in the Friedman test; according to the post hoc comparison, a significant decrease in the passive modality with respect to the active and the semi-assisted modalities was found in both measures (*p* < 0.05, see Figure 7 and Table 3).

Conversely, in the Auditory Stimuli, the Engagement demonstrated a significant difference (*p* < 0.05) between modalities (Table 3) with higher values in the music Task (Figure 7). However, NASA-TLX Overall scores were lower in the music Task than in the metronome Task (Figure 7), but this difference was not statistically significant (Table 3).

### 3.3. Objective and Subjective Measures Correlations

The analysis showed a significant moderate correlation between the AVNN and the NASA-TLX Overall (*p* = 0.010, r = −0.46) in the Auditory Stimuli (Table 4) and an almost-significant weak correlation (*p* = 0.062, r = −0.24) in the Assistance Levels (Table 5). All other comparisons resulted in negligible correlations, very far from achieving statistical significance, as shown in Table 4 and Table 5.

## 4. Discussion

In this study, we proposed a multidomain analysis combining objective and subjective measures to evaluate the modulation of MWL and engagement during upper-limb robotic-assisted movements. In particular, EEG and ECG signals were complemented with NASA-TLX and SSSQ questionnaires to comprehensively assess MWL and engagement changes at three levels of robotic assistance (active, passive and semi-assisted) and two auditory stimuli (metronome and music track).

Regarding the effects of auditory stimulation, our results showed that music-based stimuli significantly enhanced participant engagement, as indicated by both objective and subjective measures. The significant increase in EI and subjective engagement during the music task supports the idea that music enhances user involvement, even in simple, repetitive tasks, at both neurophysiological and perceptual levels. This finding is consistent with the work of Baur et al. [26], who, in a randomized study using an arm therapy robot, showed that integrating creative musical elements significantly increases intrinsic motivation, even when perceived effort or workload does not change. Similarly, Liu et al. [27] demonstrated in a pilot clinical trial that combining music therapy with robot-assisted training improves self-efficacy and positive emotion in post-stroke patients. The alignment between objective and subjective measures in our results further reinforces the idea that music should be considered as a valuable element when the goal is to enhance patient engagement during upper-limb rehabilitation.

Moreover, objective indexes did not reveal differences in MWL between music and metronome tasks; however, participants tended to perceive the music condition as slightly less demanding, as indicated by the NASA-TLX questionnaire results in Figure 7, although the decrease was not statistically significant. A plausible explanation is that more pleasant or intrinsically rewarding stimuli can support sustained involvement, thereby reducing the subjective perception of effort. This evidence suggests that motivational context can meaningfully influence how individuals experience and approach rehabilitation tasks, potentially contributing to better adherence, reduced dropout in long-term training, and ultimately enhancing the likelihood of functional recovery [21].

Although neither the NASA-TLX Overall nor the AVNN shows a significant difference between modalities, the significant negative correlation between these two measures in the Auditory Stimuli group reflects the higher perceived MWL. Accordingly, when subjects encounter situations that require higher cognitive involvement, the autonomic nervous system (ANS) modulates cardiac activity, thereby reflecting a physiological response [71].

Regarding the level of robotic assistance, we observed that subjective engagement and MWL are significantly lower in the passive modality. This result is consistent with studies showing that excessive robotic assistance can lead to “slacking” behavior, in which users reduce their effort and rely solely on the device. This can diminish effort, motivation, and perceived engagement [44,72].

Conversely, regarding the objective assessment, the EI did not reveal a statistically significant difference across modalities, although the trend was consistent with the subjective engagement. This suggests that subjective measures may capture motivational aspects that are not fully reflected in the EEG activity. This aspect, together with the non-significant and weak correlations found between objective and subjective engagement measures in Assistance Levels and Auditory Stimuli, suggests that analyzing both objective physiological signals and subjective measures provides a more complete assessment of the subject’s state, as each signal can highlight aspects that the other may miss.

Switching to the evaluation of MWL, only the AVNN was sensitive to differences in the level of assistance, revealing significantly higher values in the passive modality, indicating a decrease in ANS activity due to a higher sympathetic activation state [71]. The choice to use the AVNN was made because of time constraints as the protocol provided only 40 s of movement and HRV parameters could not be computed. Nevertheless, it was shown that this amount of time remains sufficient to assess ANS variations [73]. The discrepancy between the statistical significance among the objective MWL measures can be explained by the fact that AVNN and MWLI capture partially distinct aspects of workload. MWLI primarily reflects cortical mental effort and is therefore more closely tied to the task’s cognitive demands. In contrast, AVNN is also influenced by autonomic changes associated with motor and physical demands [74]. As a result, in the passive condition, where participants are less physically engaged, AVNN tends to increase even when cortical workload remains relatively unchanged. Therefore, its use as a pure cognitive load marker is questionable, and combining EEG- and ECG-derived markers may provide a more appropriate and comprehensive characterization of the underlying processes associated with MWL [75].

It should be noted that the selected movement (e.g., “bilateral repetitive flexo/extension of the elbow”) was chosen, among the various configurations for using the PhiCube device, as it allows bilateral action of both limbs, involving multiple joints (shoulder flexion–extension, elbow flexion–extension, wrist adduction/abduction), rather than just one joint. Moreover, this movement can be safely repeated across multiple trials, is sufficiently standardized to support meaningful physiological comparisons, is easy to learn and perform, and enables seamless integration of the three selected assistive modalities.

As concerns the auditory stimulation, it was implemented using metronome cues as a controlled baseline and music as an ecologically relevant stimulus. Indeed, music can make physical activity easier, less tiring, and more sustainable, both physically and mentally [76]. We also aimed to provide a musical stimulus that participants would likely enjoy, as musical enjoyment is a crucial moderator of music’s effects on exercise [77]. For practical reasons, it was not possible to allow each participant to choose their favorite song; therefore, we opted for a well-known song, considering that musical familiarity helps reduce interindividual variability in enjoyment [77,78]. Furthermore, the literature indicates that a stable beat reduces movement temporal variability and facilitates motor entrainment [24]. For this reason, we selected a song with a constant BPM, particularly suited to continuous rhythmic tasks. Considering all the mentioned aspects, among the various possible options, we chose “Stayin’ Alive” by the Bee Gees.

The absence of significant changes in EEG-derived MWLI across conditions may primarily reflect the characteristics of the task paradigm rather than true invariance in cognitive workload. In particular, the motor task adopted in this study is simple, highly repetitive, and performed by healthy participants; once familiarized, it is likely to rely largely on automatic sensorimotor control and therefore imposes a relatively low cognitive demand. Under these conditions, the modulation of mental workload induced by different assistance levels may be too subtle to elicit measurable changes in the selected EEG-based MWLI, or may be confined to short, transient phases (e.g., initial adaptation or occasional corrections) that are attenuated when computing indices over longer epochs. However, it should be considered that patients affected by upper limb impairment may experience considerable difficulty even in performing simple movements, potentially leading to higher MWL and clearer differences between passive, assisted and active modalities.

To the best of our knowledge, no studies have specifically evaluated the MWLI associated with the level of robot assistance in the rehabilitation context. Nevertheless, this result shows that measures derived from physiological signals, which are often assumed to capture the same dimensions, can differ in sensitivity because they reflect partially different aspects. This highlights the value of acquiring and analyzing physiological indices from multiple signals, as it provides a more complete picture of the patient’s state, without overcomplicating the setup.

Overall, our findings highlight the value of considering multiple objective physiological signals and subjective scales to characterize user experience and state during robot-assisted rehabilitation. Although simplifying acquisition protocols by reducing the number of data acquired can help minimize preparation time and, consequently, the burden on the patient, our results also show that collecting multiple physiological signals and psychological measures together provides a more comprehensive assessment of the subject’s overall state during the rehabilitation session. The combined use of EEG, ECG, and validated psychometric tools proved feasible and informative, revealing complementary facets of workload and engagement under different assistance and motivational conditions.

## 5. Limitations and Future Work

Some limitations should be acknowledged before discussing the future development of the findings. The first is the reduced sample size, and the second is the inclusion of only healthy participants, which limits the generalization of the findings. Although this choice ensured strict experimental control, it may also explain the absence of significant differences between the AME and SME modalities in all physiological and subjective parameters and the non-significant differences in MWLI across assistance levels. In clinical populations with motor impairments, these conditions are likely to differ more markedly in terms of required effort, and such differences may be more readily captured by the analyzed measures.

The multimodal analysis explored in our work opens promising avenues for adaptive rehabilitation systems that dynamically modulate robotic assistance and motivational content based on real-time psychophysiological feedback. This approach could help maintain engagement, prevent overload and monotony, and ultimately support better adherence and functional outcomes over the long term of training. Future research could test the proposed framework with individuals with motor impairment. This would determine whether different levels of assistance affect MWL and engagement during robotic rehabilitation in people with disabilities.

Moreover, a limited number of experimental variations was implemented to reduce session complexity and to preserve data interpretability by isolating the primary factors of interest. Therefore, future studies should also evaluate additional movement families and music characteristics (genre, tempo, personalization) to determine whether the observed effects generalize beyond the controlled conditions tested here.

To further enrich the cognitive/psychological assessment, testing longer task durations could help clarify whether prolonged execution of repetitive movements leads to decreased motivation due to boredom or fatigue, thereby negatively affecting MWL and user engagement. In this context, longer periods of movement within a single session can be exploited to compute HRV parameters (e.g., Low Frequency/High Frequency (LF/HF), RMSSD, and SDNN), which require longer recording windows to be reliably estimated. These measures could offer richer insights into autonomic modulation across conditions. Hence, exploring different durations of tasks may be necessary to identify an optimal trade-off between recordings long enough to ensure reliable physiological estimates and rehabilitation sessions that remain effective, engaging and not too demanding for the patient.

## 6. Conclusions

This study shows that a multimodal assessment combining objective physiological measures (EEG and ECG) with subjective reports can capture distinct aspects of MWL and engagement during upper-limb robot-assisted rehabilitation. While the EEG-derived MWLI did not show differences across assistance levels, subtle changes in MWL across conditions were detected by AVNN (ECG-derived). However, this likely reflects variations in autonomic activation associated with physical engagement, rather than solely cortical MWL, highlighting the importance of acquiring different physiological signals that can evidence distinct aspects of the same concept. In addition, EEG-derived EI did not differ significantly across assistance levels. Conversely, perceived MWL and engagement were significantly lower in the passive modality compared with the active and semi-assisted conditions despite no detectable changes in the objective EEG indexes, highlighting that self-reports can reveal motivational/experiential components not captured by EEG alone. Finally, music significantly increased perceived engagement and the EEG-derived EI without significantly affecting MWL, suggesting that auditory stimulation may improve the rehabilitation experience without increasing cognitive demand.

Overall, these results support the use of complementary objective–subjective metrics to guide the personalization of robot-assisted rehabilitation protocols. Future work should apply this framework to clinical populations with motor impairments to determine whether assistance levels and music similarly modulate MWL and engagement, and whether these factors translate into improved adherence and outcomes.

## Figures and Tables

**Table 1:** Characterization by age group of the population. SD stands for the standard deviation of each group’s age in years.

Number of Subjects	Age (Years)	Mean Age	SD
17	18–30	25.4	2.3
8	31–50	40.9	5.6
5	51–65	55.2	5.4

**Table 2:** Nemenyi (for Assistance Levels) and Wilcoxon (for Auditory Stimuli) results for every objective index analyzed. The minus sign (−) indicates that no Nemenyi post hoc test was conducted, given the non-significant difference observed in the results of the Friedman test.

OBJECTIVE INDEX	Assistance Levels			Auditory Stimuli
	**AME-SME**	**SME-PME**	**AME-PME**	**AME-AMU**
MWLI	−	−	−	*p* = 0.746
EI	−	−	−	*p* < 0.001 *
AVNN	0.863	0.004 *	*p* < 0.001 *	*p* = 0.158

* Statistically significant (α < 0.05).

**Table 3:** Nemenyi (for Assistance Levels) and Wilcoxon (for Auditory Stimuli) results for every subjective index analyzed.

SUBJECTIVE INDEX	Assistance Levels			Auditory Stimuli
	**AME-SME**	**SME-PME**	**AME-PME**	**AME-AMU**
NASA-TLX Overall	*p* = 0.991	*p* = 0.015 *	*p* = 0.022 *	*p* = 0.203
Engagement	*p* = 0.638	*p* = 0.012 *	*p* < 0.001 *	*p* = 0.023 *

* Statistically significant (α < 0.05).

**Table 4:** Repeated measures correlation results for Auditory Stimuli. The *p*-value and the correlation coefficient (r) are reported for each correlation. The minus sign (−) indicates that no correlation was performed.

	NASA-TLX Overall	Engagement
**MWLI**	*p* = 0.573, r = 0.11	−
**EI**	−	*p* = 0.571, r = 0.11
**AVNN**	*p* = 0.010 *, r = −0.46	−

* Statistically significant (α < 0.05).

**Table 5:** Repeated measures correlation results for Assistance Levels. The *p*-value and the correlation coefficient (r) are reported for each correlation. The minus sign (−) indicates that no correlation was performed.

	NASA-TLX Overall	Engagement
**MWLI**	*p* = 0.694, r = 0.05	−
**EI**	−	*p* = 0.438, r = 0.1
**AVNN**	*p* = 0.062, r = −0.24	−

## Data Availability

The raw data supporting the conclusions of this article will be made available by the authors upon request.

## Abbreviations

The following abbreviations are used in this manuscript:

MWL	Mental Workload
EEG	Electroencephalography
ECG	Electrocardiogram
NASA-TLX	NASA Task Load Index
SSSQ	Short Stress State Questionnaire
HRV	Heart Rate Variability
MWLI	Mental Workload Index
GUI	Graphical User Interface
AME	Active with Metronome
AMU	Active with Music
PME	Passive with Metronome
SME	Semi-assisted with Metronome
EI	Engagement Index
AVNN	Average Value of Normal-to-Normal Intervals
ANS	Autonomic Nervous System
LF/HF	Low Frequency/High Frequency
RMSSD	Root Mean Square of Successive Differences
SDNN	Standard Deviation of Normal-to-Normal Intervals
